# Low‐Density Lipoprotein Cholesterol Increases Significantly During Brief Discontinuation of Atorvastatin and Correlates With Metabolite Half‐Lives

**DOI:** 10.1002/prp2.70082

**Published:** 2025-03-26

**Authors:** Jonas Pivoriunas, Nils Tore Vethe, Oscar Kristiansen, Stein Bergan, Einar Husebye, John Munkhaugen, Elise Sverre

**Affiliations:** ^1^ Department of Medicine Drammen Hospital, Vestre Viken Hospital Trust Drammen Norway; ^2^ Department of Behavioural Medicine, Institute of Basic Medical Sciences, Faculty of Medicine University of Oslo, Domus Medica Oslo Norway; ^3^ Department of Pharmacology Oslo University Hospital Oslo Norway; ^4^ Department of Pharmacy University of Oslo Oslo Norway

**Keywords:** adherence, atorvastatin, atorvastatin metabolites, lipids, liquid chromatography–tandem mass spectrometry, statins

## Abstract

Variability in low‐density lipoprotein cholesterol (LDL‐C) has emerged as a potential independent cardiovascular risk factor, but the impact of short‐term discontinuation of statins on LDL‐C remains to be defined. Furthermore, the relationship between individual statin metabolites and changes in LDL‐C has not yet been examined. The present study aimed to investigate changes in LDL‐C concentrations during a four‐day discontinuation of atorvastatin therapy and to examine correlations between the half‐lives of atorvastatin metabolites and LDL‐C concentrations. This pharmacokinetic intervention study included 60 adults with confirmed adherence to atorvastatin, using doses of 20 mg (*N* = 20), 40 mg (*N* = 20), or 80 mg (*N* = 20) at study start. Atorvastatin was then discontinued, and blood samples were collected from day zero to day four. We assessed daily concentrations of LDL‐C and of atorvastatin with its metabolites by liquid chromatography–tandem mass spectrometry. The mean (SD) LDL‐C at baseline was 1.84 (0.6) mmol/L. LDL‐C increased on average by 0.50 mmol/L (27%) from day zero to day four. The increase in LDL‐C was significant already 48 h after the last statin intake and was affected by individual variation in baseline concentrations and the slope of the daily increase. A moderate correlation was found between differences in LDL‐C concentrations and the half‐lives of hydroxylated atorvastatin metabolites. In conclusion, 4 days without atorvastatin resulted in an almost 30% increase in LDL‐C concentrations, and the increase was significant already after the first omitted dose. The half‐lives of hydroxylated atorvastatin metabolites showed a moderate correlation with the increase in LDL‐C concentrations.

## Graphical abstract

Created in BioRender. Munkhaugen, J. (2025) https://BioRender.com/g40l253.

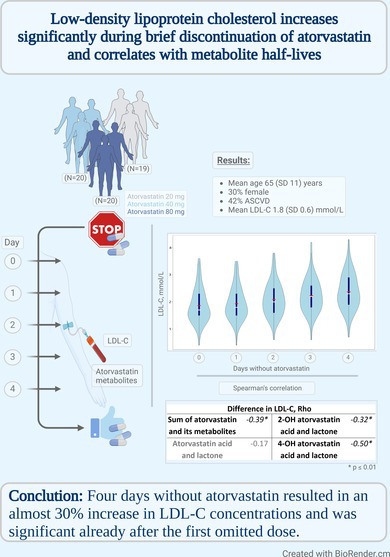

## 1 Introduction

Statins efficiently reduce low‐density lipoprotein cholesterol (LDL‐C) concentrations and decrease the risk of atherosclerotic cardiovascular disease (ASCVD) [1]. There are, however, substantial individual variations in LDL‐C lowering effects across all statin classes and doses [2]. Reduced drug adherence has been proposed as a major cause of inadequate response to statin therapy associated with unfavorable LDL‐C control and subsequent CV events [3, 4].

Although some clinical and genetic factors have been associated with LDL‐C response following statin treatment, most of the inter‐ and intra‐individual variation in LDL‐C remains unexplained [2, 5]. Visit‐to‐visit variability in LDL‐C is an independent determinant associated with adverse outcomes in patients with ASCVD [6, 7, 8]. The association between LDL‐C variability and prognosis is hypothesized to be caused primarily by atherosclerosis, but also by the nonlipid pleiotropic effects of statins [6, 9]. A better understanding of variations in LDL‐C concentrations during short‐term statin discontinuation is therefore required.

Data on the relationship between atorvastatin metabolite concentrations and LDL‐C concentrations are limited. In a post hoc analysis of a randomized trial, we recently assessed the relationship between atorvastatin metabolite concentrations and LDL‐C response on a fixed dose of 40 mg daily [10]. However, metabolite concentrations explained only 14% of the total variation in LDL‐C, with a high degree of uncertainty. Longitudinal measurements of LDL‐C and atorvastatin metabolites were not conducted in that study [10].

The objective of the present study was to examine changes in LDL‐C concentrations in blood plasma after short‐term discontinuation of atorvastatin, defined by one to 4 days off treatment. Additionally, we studied the relationship between changes in LDL‐C concentrations and the half‐lives of atorvastatin and its major metabolites, using a novel assay to quantify these concentrations directly in blood plasma by liquid chromatography–tandem mass spectrometry (LC–MS/MS) [11].

## 2 Methods

### 2.1 Study Design

**FIGURE 1:**
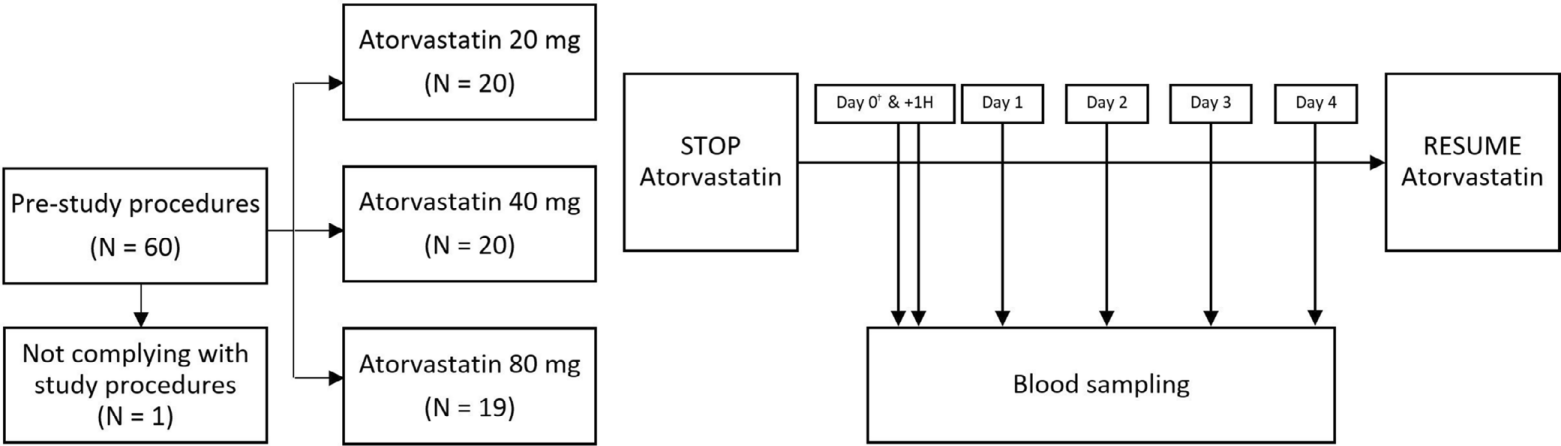
Flowchart of the study. †24 h after the last atorvastatin dose.

Sixty participants, both with and without ASCVD, were recruited from the outpatient clinic at Drammen Hospital, Norway, for a pharmacokinetic intervention study conducted between February and May 2021. One participant did not adhere to the study protocol and was subsequently excluded, resulting in a final sample size of 59 participants. A total of 57 participants were on long‐term atorvastatin therapy for primary or secondary prevention, while three participants had initiated atorvastatin therapy less than 3 months prior to the start of the study. We enrolled 20 participants using atorvastatin 20 mg/day, 20 participants using atorvastatin 40 mg/day, and 19 using atorvastatin 80 mg/day. All participants with ASCVD were recruited at least 2 years post‐event and were in stable condition (Figure 1).

### 2.2 Study Procedures

Prior to the study start, eligible candidates attended a one‐hour session with study physicians and nurses at the outpatient clinic. Comprehensive information about the study protocol was provided, and informed consent was obtained. Prior to the study start, participants were instructed to take atorvastatin consistently in the morning (between 7 and 10 a.m.) under regular conditions for at least 1 week and encouraged to record the precise time of atorvastatin intake in a medication diary. To assure a steady state for atorvastatin metabolite concentrations in blood, the first sampling of blood was scheduled 24 h after the last atorvastatin intake. Participants were instructed not to change their eating habits or medications during the study period. Following this phase, atorvastatin was ceased from day zero to day four. The day of the first blood sample for atorvastatin, its five major metabolites, and LDL‐C is referred to as day zero. To validate adherence to the study protocol and ensure no unscheduled atorvastatin doses were taken, an additional blood sample was obtained 25 h after the last intake of atorvastatin. Subsequently, blood samples were drawn at the same time each morning from day one to day four. Atorvastatin was restarted after the blood sample on day four if indicated.

### 2.3 Clinical Data

Demographic and clinical data were collected from the hospital records and included: age, sex, body weight, somatic comorbidities (ASCVD, atrial fibrillation, diabetes mellitus hypertension, and heart failure), use of nonstatin lipid‐lowering treatment, and other medications. Daily laboratory measurements were performed to obtain the lipid profile: total cholesterol, LDL‐C, high‐density lipoprotein cholesterol (Alinity, Abbot Laboratories, Abbot Park, IL, USA). Daily measurements of atorvastatin and its major metabolites (the acid and lactone forms of atorvastatin, ortho‐(2‐OH)‐hydroxyl atorvastatin, and para‐(4‐OH) hydroxyl atorvastatin) were assessed using a two‐channel LC–MS/MS assay (Thermo Fisher Scientific, Waltham, MA, USA) at Oslo University Hospital [12].

### 2.4 Assessment of Changes in LDL‐C Concentrations

During the atorvastatin discontinuation period, we explored the absolute and relative changes in LDL‐C concentrations, the effect of individual LDL‐C concentrations at baseline (day zero), the individual slope of the daily increase in LDL‐C concentrations, and the potential effect of the atorvastatin dose.

### 2.5 Assessment of Atorvastatin and Metabolites Concentrations

Lactone forms of atorvastatin and its major metabolites undergo continuous conversion to their acid forms at ambient temperature [11]. The NORwegian CORonary (NORCOR) research group has developed a methodology to assess the sums of the corresponding lactone–acid pairs (atorvastatin, 2‐OH atorvastatin and 4‐OH atorvastatin), ensuring the stability of measurements for 6 days at ambient temperature [11]. The present study analyzed the relationship between the difference in LDL‐C concentrations during atorvastatin discontinuation and the half‐lives of the sum of atorvastatin and its major metabolites, and corresponding lactone–acid pairs.

### 2.6 Statistical Analysis

We described the study participants using mean and standard deviation for continuous variables and percentages for categorical values. Violin plots were used to visualize the distribution of LDL‐C concentrations across days during atorvastatin discontinuation, including kernel density estimation, maximal and minimal interquartile range, means, and medians of LDL‐C values. To illustrate individual changes in LDL‐C concentrations during atorvastatin discontinuation, participants were randomly divided into four groups for visual illustration only.

To model LDL‐C concentrations by the duration of atorvastatin discontinuation, we applied a mixed‐effects model (using restricted maximum likelihood (REML) estimation). This model provides the possibility to account for the known and expected individual variation [2] in both baseline LDL‐C concentrations (variability of LDL‐C concentrations at day zero) and the increase in LDL‐C over time of discontinuation (variability in the slope of the daily increase). The increase in LDL‐C concentrations was approximated by a linear curve with a good fit to the observed data. The impact of atorvastatin dose was also evaluated.

We used Spearman's rank test to correlate changes in daily LDL‐C concentrations with the half‐lives of the sum of atorvastatin and its major metabolites, including lactone–acid metabolite pairs (2‐OH atorvastatin and 4‐OH atorvastatin). All statistical analyses were performed using R version 2023.12.1 and Stata version 17.

### 2.7 Ethics and Safety

The protocol of the present study was approved by the Regional Committee of Medical and Health Research Ethics (number 197069) and the local Data Protection Officer (number 21/01282–1). The study has been carried out in accordance with the Declaration of Helsinki. Short‐term discontinuation of statin therapy in stable CVD patients is assumed to be safe [13].

## 3 Results

Among the 59 study participants, the mean age was 65 (SD 11) years, 18 (30%) were female, and 25 (42%) had ASCVD (Table 1). Participants had an average of 4.9 measurements of LDL‐C and atorvastatin metabolites concentrations.

**TABLE 1:** Characteristics of participants at study start.

Characteristic	Total (*N* = 59)
Age (years), mean (SD)	65.4 (11.2)
Male, *n* (%)	41 (70)
Body weight (kg), mean (SD)	87.2 (16.6)
ASCVD, *n* (%)	25 (42)
Atrial fibrillation, *n* (%)	22 (37)
Diabetes mellitus, *n* (%)	10 (17)
Hypertension, *n* (%)	22 (37)
Heart failure, *n* (%)	20 (34)
Use of ezetimibe, *n* (%)^a^	13 (22)
Total C (mmol/L)^b^, mean (SD)	3.5 (0.9)
LDL‐C (mmol/L)^b^, mean (SD)	1.8 (0.6)
HDL‐C (mmol/L)^b^, mean (SD)	1.1 (0.3)

Abbreviations: ASCVD, arteriosclerotic cardiovascular disease; HDL‐C, high‐density lipoprotein cholesterol; LDL‐C, low‐density lipoprotein cholesterol; SD, standard deviation; Total C, total cholesterol.

^a^ No other non‐statin lipid‐lowering treatment was used.

^b^ Measured at day zero.

### 3.1 Changes of LDL‐C Concentrations in Blood

**FIGURE 2:**
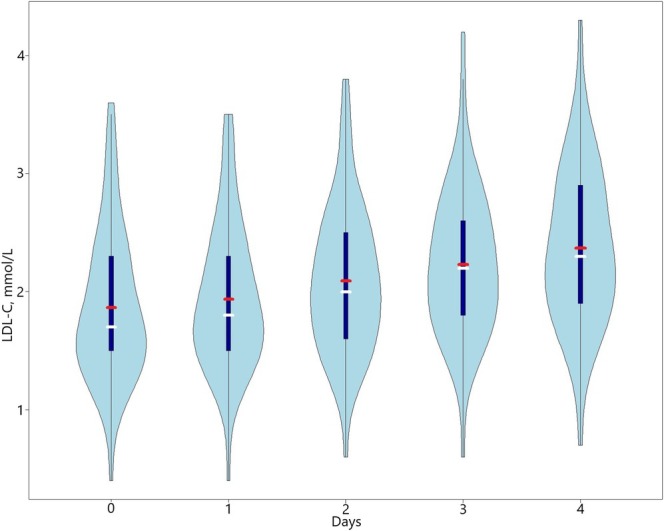
Violin plot of low‐density lipoprotein cholesterol concentrations from day zero to day four during atorvastatin discontinuation. The width of each violin plot represents the kernel density estimate (KDE) of low‐density lipoprotein cholesterol (LDL‐C) measurements at different concentrations, with the tips indicating the minimal and maximal values in the estimate. The red horizontal line indicates the mean LDL‐C concentration, while the white horizontal line indicates the median. The thick dark blue vertical line shows the interquartile range from the 25th (first quartile) to the 75th percentile (third quartile).

**FIGURE 3:**
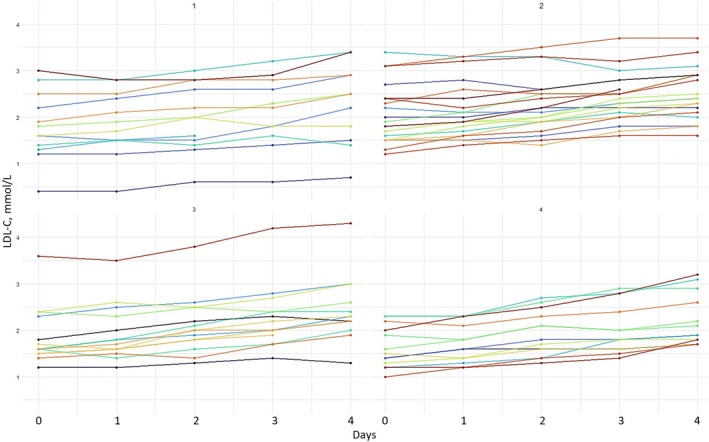
The individual increase of low‐density lipoprotein cholesterol concentrations in four random groups. Each line represents an individual participant's low‐density lipoprotein cholesterol measurements from day zero to day four during atorvastatin discontinuation. Participants were randomly divided into four groups for visual illustration only. LDL‐C, low‐density lipoprotein cholesterol.

LDL‐C concentrations increased on average by 0.50 mmol/L (SD 0.04), representing a 27% increase from day zero to day four. All but one participant (98%) had an increase in LDL‐C concentrations, regardless of the atorvastatin dose. The variability of LDL‐C concentrations at day zero and the increase in LDL‐C concentrations during atorvastatin discontinuation are shown in Figures 2 and 3. The increase was statistically significant already after the first omitted dose, with a predicted daily linear increase in LDL concentrations of 0.13 mmol/L (95% CI 0.11–0.15). There was significant individual variability in LDL‐C concentrations both at day zero (SD 0.60; 95% CI 0.50–0.73) and in the slope of the daily increase (SD 0.06; 95% CI 0.05–0.07) (Table 2).

**TABLE 2:** Mix‐models summary for low‐density lipoprotein cholesterol concentrations.

	Population averages (fixed effects)		Individual variation (random effects)	
	Estimated mean, mmol/L	95% CI	Standard deviation	95% CI for SD
LDL‐C concentrations at day zero	1.84	1.68–2.00	0.6	0.50–0.73
Slope of daily increase of LDL‐C	0.13	0.11–0.15	0.06	0.05–0.07

Abbreviations: CI, confidence interval; LDL‐C, low‐density lipoprotein cholesterol; SD, standard deviation.

At a higher atorvastatin dose, there was a tendency for lower LDL‐C concentrations 24 h after the last dose and somewhat faster increasing daily LDL concentrations during atorvastatin discontinuation (Figure SS1) not reaching statistical significance (data not shown). Additional use of ezetimibe did not affect the daily increase in LDL‐C (data not shown).

### 3.2 Atorvastatin Metabolites

**FIGURE 4:**
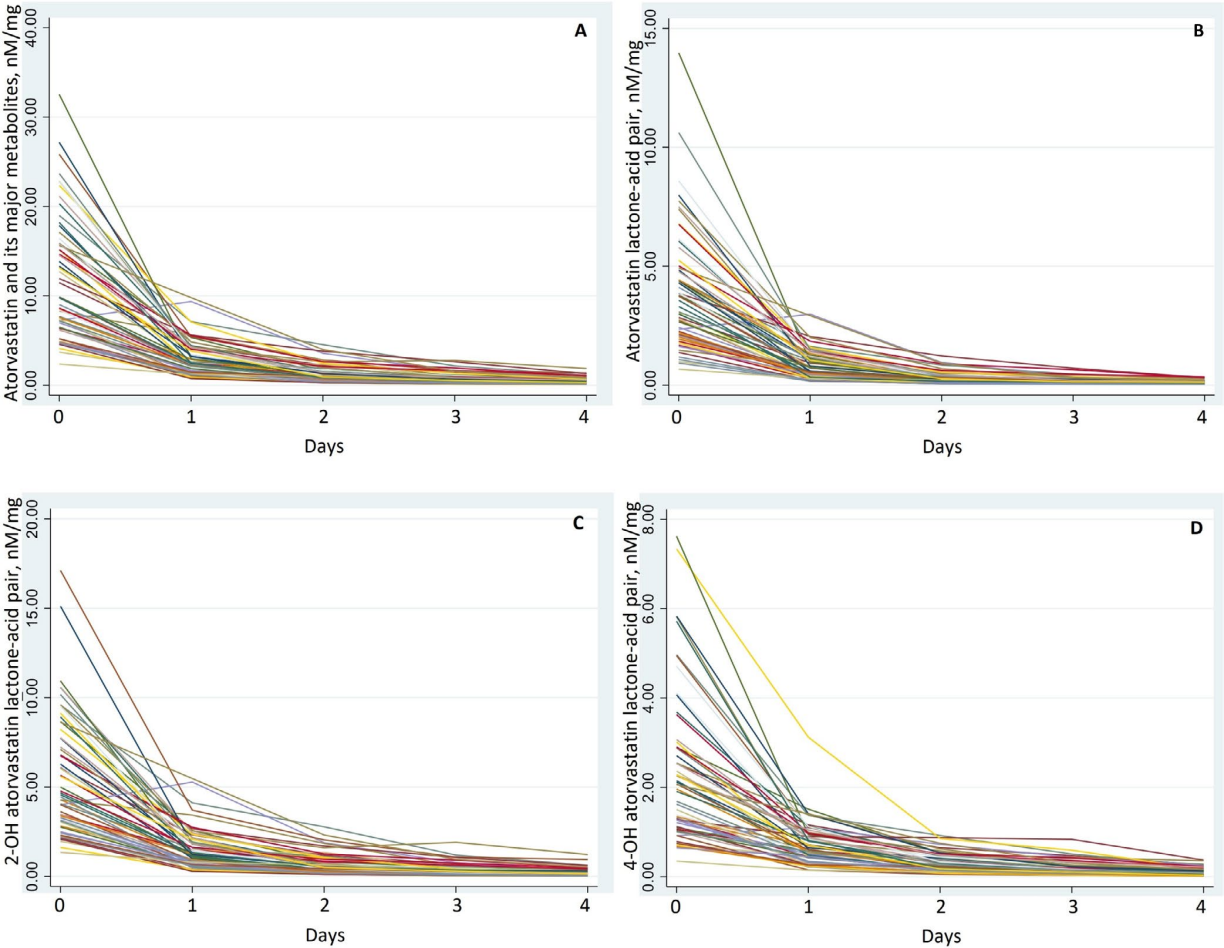
Change in atorvastatin metabolites concentrations from day zero to day four. (A) Change in atorvastatin and its major metabolites concentrations from day zero to day four, (B) Change in atorvastatin lactone‐acid pair concentrations from day zero to day four, (C) Change in 2‐OH atorvastatin (ortho‐hydroxyl) atorvastatin lactone‐acid pair concentrations from day zero to day four, (D). Change in 4‐OH atorvastatin (para‐hydroxyl) atorvastatin lactone‐acid pair concentrations from day zero to day four.

The changes in atorvastatin metabolites concentrations showed a rapid nonlinear decrease from day zero to day one, followed by a flattening of the curves (Figure 4).

The half‐life of the sum of atorvastatin and its major metabolites was 20.6 h (SD 4.5; 95% CI 19.4–21.7). For the corresponding lactone–acid pair, the half‐life was 19.7 h (SD 4.0; 95% CI 18.7–20.7) for atorvastatin, 20.4 h (SD 5.3; 95% CI 19.0–21.7) for 2‐OH atorvastatin, and 23.1 h (SD 7.9; 95% CI 21.0–25.1) for 4‐OH atorvastatin.

There was a moderate correlation between the change in LDL‐C concentrations from day zero to day four and the half‐lives of the sum of atorvastatin and its major metabolites, as well as the 2‐OH atorvastatin lactone–acid and 4‐OH atorvastatin lactone–acid pairs. However, the correlation with the atorvastatin lactone–acid pair was weak and not statistically significant (Table 3 and Figure SS2).

**TABLE 3:** Spearman's correlation between low‐density lipoprotein cholesterol concentrations and half‐lives of atorvastatin metabolites.

	Sum of atorvastatin and its metabolites	Atorvastatin acid and lactone	2‐OH atorvastatin acid and lactone	4‐OH atorvastatin acid and lactone
Difference in LDL‐C concentrations, rho	−0.39 *p = 0.002*	−0.17 *p = 0.19*	−0.32 *p = 0.01*	−0.50 *p < 0.001*

Abbreviations: 2‐OH atorvastatin, ortho‐hydroxyl atorvastatin; 4‐OH atorvastatin, para‐hydroxyl atorvastatin; LDL‐C, low‐density lipoprotein cholesterol; rho, Spearman's rank correlation coefficient.

## 4 Discussion

This pharmacokinetic intervention study showed that discontinuing any clinically relevant dose of atorvastatin for just 4 days significantly elevates LDL‐C concentrations in blood, with a total increase of 27% on average. The average daily increase in LDL‐C concentrations was 0.13 mmol/L and was significant after the first omitted dose. We found considerable individual variations in both day zero LDL‐C concentrations and the slope of the daily LDL‐C increase. The half‐lives of hydroxylated atorvastatin metabolites showed a moderate correlation with the increase in LDL‐C.

Dynamic changes in physiological parameters are essential components of homeostasis, and the intra‐individual variation in LDL‐C likely reflects the complexity of cholesterol metabolism. A biological, bidirectional intra‐individual variation of 8.5% in LDL‐C measurements is expected in healthy volunteers [14]. However, the greater unidirectional increase of LDL‐C concentrations observed in the present study suggests that the pharmacodynamic effect of atorvastatin discontinuation on LDL‐C concentrations exceeds the aforementioned biological mechanisms [14]. Additionally, a small study of ten male volunteers with metabolic syndrome and without other chronic or CV diseases reported a similar LDL‐C increase rate of 0.13 mmol/L from day four to day 15 after statin discontinuation [15].

“Spot” sampling is influenced by many variables, such as prior LDL‐C concentrations, statin adherence [16], diet, genetic variation, and other factors that can affect these measurements [5]. However, “spot” measurements are directly related to cardiovascular risk and influence clinical decision‐making, such as intensifying lipid‐lowering therapy if treatment targets are not met. In turn, this may increase the risk of adverse effects with higher statin doses and potentially lead to higher treatment expenses [17]. The rapid individual increase in LDL‐C concentrations observed after only 1 day without statin emphasizes an additional important limitation in the accuracy of a single LDL‐C measurement for therapy decisions. Awareness should be taken in clinical practice to identify patients at risk of nonadherent behavior by assessing drug adherence when LDL‐C is measured [18].

LDL‐C variability over time is independently related to increased ASCVD risk [2, 6]. Bangalore et al. demonstrated that each standard deviation increase in LDL‐C variability is associated with a 10%–20% increased risk of any cardiovascular event, and the increased risk remained even after adjusting for reduced statin adherence [6]. Other studies have shown that LDL‐C variability increases the risk of adverse cardiovascular and cerebrovascular events by 37% [8, 19], and accelerates coronary atheroma progression [20]. A nonlipid, but so‐called pleiotropic, mechanism is suggested as a cause of this risk [6]. In the present study, the LDL‐C concentrations significantly increased after just one omitted dose. The effects of LDL‐C variability due to repetitive short‐term statin discontinuation are not well studied. Rapid initiation of statins in patients with acute coronary syndrome has shown beneficial outcomes with regard to major adverse cardiac events [21], and positive effects on coronary flow have also been observed just 1 h after atorvastatin initiation [22]. The growing evidence of the rapid beneficial effects of statins preceding LDL‐C lowering [23] raises a question about the harm of short‐term repetitive discontinuation of statin therapy. Such inconsistent adherence to prescribed medication may pose a previously unidentified risk to patients [6, 7]. It is likely that interventions aimed at ensuring patient adherence to prescribed medications represent the most feasible modification that could be implemented at this time. However, reduced adherence is common in clinical practice, and an accurate method for assessing adherence is needed [24].

The present study demonstrated a moderate correlation between the half‐life measurements of hydroxylated atorvastatin metabolites and the increase in LDL‐C concentrations, suggesting a more direct association between these metabolites and the effect of atorvastatin on LDL‐C concentrations. Additionally, hydroxylated forms are reported to account for 70% of the pharmacological activity of atorvastatin [25]. These forms have prolonged half‐lives, possibly enhancing atorvastatin's LDL‐C lowering effect and, in turn, reducing the risk of major cardiovascular events [1]. A recent study by Sverre et al. also showed that 4‐OH atorvastatin lactone and acid pair provided the most sensitive estimate for the percentage reduction in LDL‐C after atorvastatin initiation, but these metabolites could only explain 14% of the total LDL‐C variation in unadjusted analysis [10]. In addition, participants with shorter metabolite half‐lives (i.e., faster drug elimination) may experience a larger increase in LDL‐C during short‐term atorvastatin discontinuations. The half‐lives of the individual atorvastatin metabolites may also affect the overall change in LDL‐C differently. Due to spontaneous interconversion between lactone and acid forms at room temperature [11], assessing separate metabolites is not futile. Both laboratory and intra‐personal LDL‐C measurements could introduce random noise, affecting the collected data [14]. Other mechanisms of statin therapy, such as pleiotropic effects, could play an important role in the treatment effect. The relationship between these pleiotropic effects, LDL‐C variability, and atorvastatin metabolites remains unknown.

### 4.1 Strengths and Limitations

The major strengths of the present study include strict control of all study variables, confirmed adherence to the study protocol, and a standardized sampling procedure and analysis.

However, potential limitations should be acknowledged. This exploratory study has a modest sample size. The sample included participants undergoing statin treatment for both primary and secondary prevention, as would be expected in the general cardiac outpatient population. All study participants were Caucasian, and interfaces would be confined to this population. The majority of the participants were on long‐term atorvastatin therapy, while three participants had initiated treatment less than 3 months prior to the study. Statin naive measurements of LDL‐C concentrations prior to study inclusion were not available. There are several statins on the market, but only the discontinuation of atorvastatin, the most frequently prescribed statin in Norway [4], was assessed in the present study. Detailed information on dietary intake was not collected, but participants were instructed to maintain their usual diet and refrain from changing their medication throughout the study period.

Women were underrepresented, comprising one‐third of the study population. The effect of atorvastatin dose on LDL‐C increase after discontinuation did not reach statistical significance, and the result could differ in a larger sample or if the dose allocation had been randomized. However, no association between the statin dose increase and LDL‐C concentrations was found in another small study [15]. The difference in timeframes used for correlating changes in LDL‐C concentrations (days) and metabolites half‐lives (hours) could potentially oversimplify the relationship between atorvastatin pharmacokinetics and the biological processes affecting LDL‐C changes.

The present study provides deeper insight into LDL‐C changes during short‐term discontinuation of atorvastatin. Long‐term observations have shown that higher LDL‐C variability is an independent risk factor for future ASCVD events [6, 7, 19]. The increase in LDL‐C after short‐term atorvastatin discontinuation may have clinical consequences, including negative effects on cardiovascular outcomes, and should be further assessed in future studies.

## 5 Conclusions

Four days of atorvastatin discontinuation resulted in an almost 30% increase in LDL‐C concentrations, which was significant already 1 day after the first omitted dose. Half‐lives of hydroxylated atorvastatin metabolites showed a moderate correlation with the increase in LDL‐C concentrations.

## Author Contributions

**Jonas Pivoriunas:** conceptualization, methodology, software, formal analysis, writing – original draft, writing – review and editing, visualization. **Nils Tore Vethe:** conceptualization, methodology, investigation, resources, writing – review and editing, supervision. **Oscar Kristiansen:** conceptualization, resources, writing – review and editing. **Stein Bergan:** conceptualization, writing – review and editing. **Einar Husebye:** conceptualization, methodology, writing – review and editing. **John Munkhaugen:** conceptualization, methodology, investigation, resources, writing – review and editing, supervision, project administration, funding acquisition. **Elise Sverre:** conceptualization, methodology, investigation, resources, writing – review and editing, supervision, project administration.

## Conflicts of Interest

J.P. reports having received a modest lecture fees from AstraZeneca, outside the submitted work. J.M. reports having received modest lecture fees from Novartis, Bayer, Sanofi, and Boehringer‐Ingelheim, outside the submitted work. N.T.V. reports having received a modest lecture fee from Novartis, outside the submitted work. No other conflicts of interest were reported.

## Supporting information


**Figure S1.** The increase of low‐density lipoprotein cholesterol concentrations by dose LDL‐C: low‐density lipoprotein cholesterol.

**Figure S2.** Scatter plots with correlation of low‐density lipoprotein cholesterol concentrations and half‐lives of different atorvastatin metabolites In each scatter plot the black dot indicates the relationship of individual observation, the regression line is a blue line. 2‐OH atorvastatin: ortho‐hydroxyl atorvastatin, 4‐OH atorvastatin: para‐hydroxyl atorvastatin, R: Spearman’s rank correlation coefficient.

## Data Availability

The data that support the findings of this study are available from the corresponding author upon reasonable request.
