# Supplementary figures and images for: Low‐Density Lipoprotein Cholesterol Increases Significantly During Brief Discontinuation of Atorvastatin and Correlates With Metabolite Half‐Lives
Source: Pharmacol Res Perspect. 2025 Mar 26;13(2):e70082. doi: 10.1002/prp2.70082 (PMC11946918; doi:10.1002/prp2.70082)

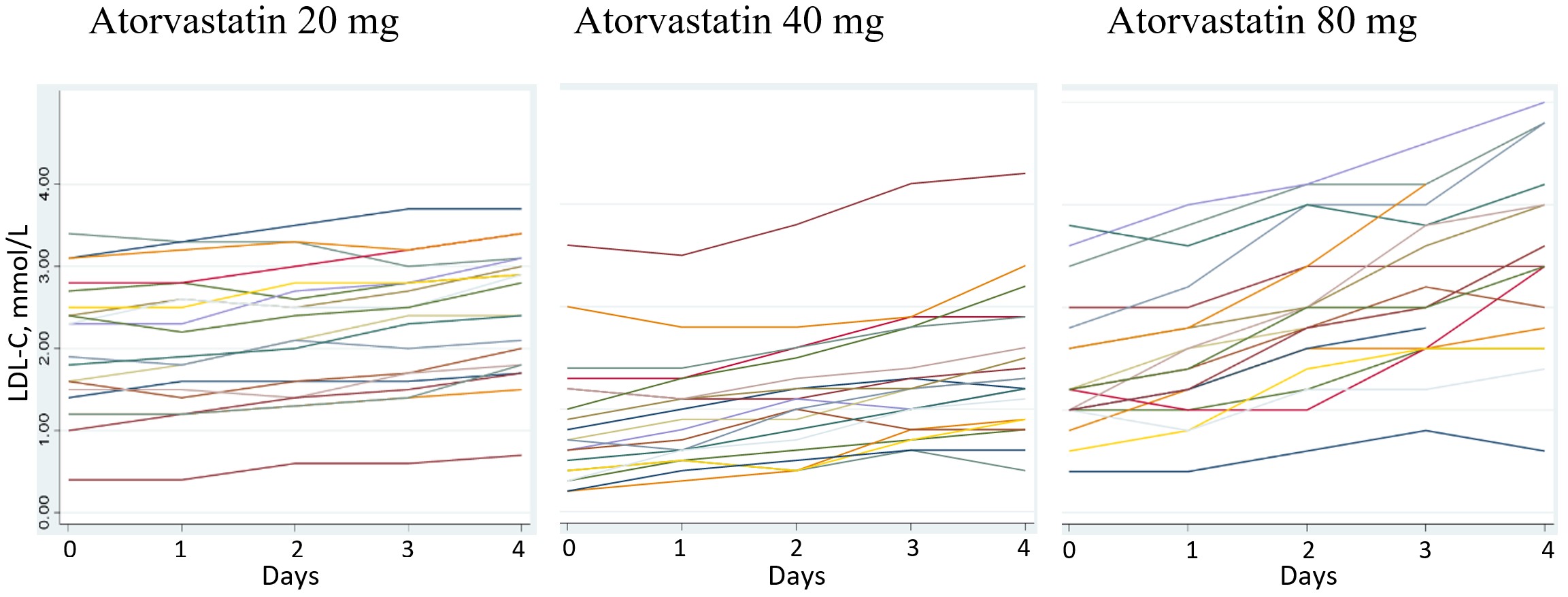

Supplement: Supplementary file 1 — Figure S1. The increase of low‐density lipoprotein cholesterol concentrations by dose LDL‐C: low‐density lipoprotein cholesterol. [file PRP2-13-e70082-s001.jpg]

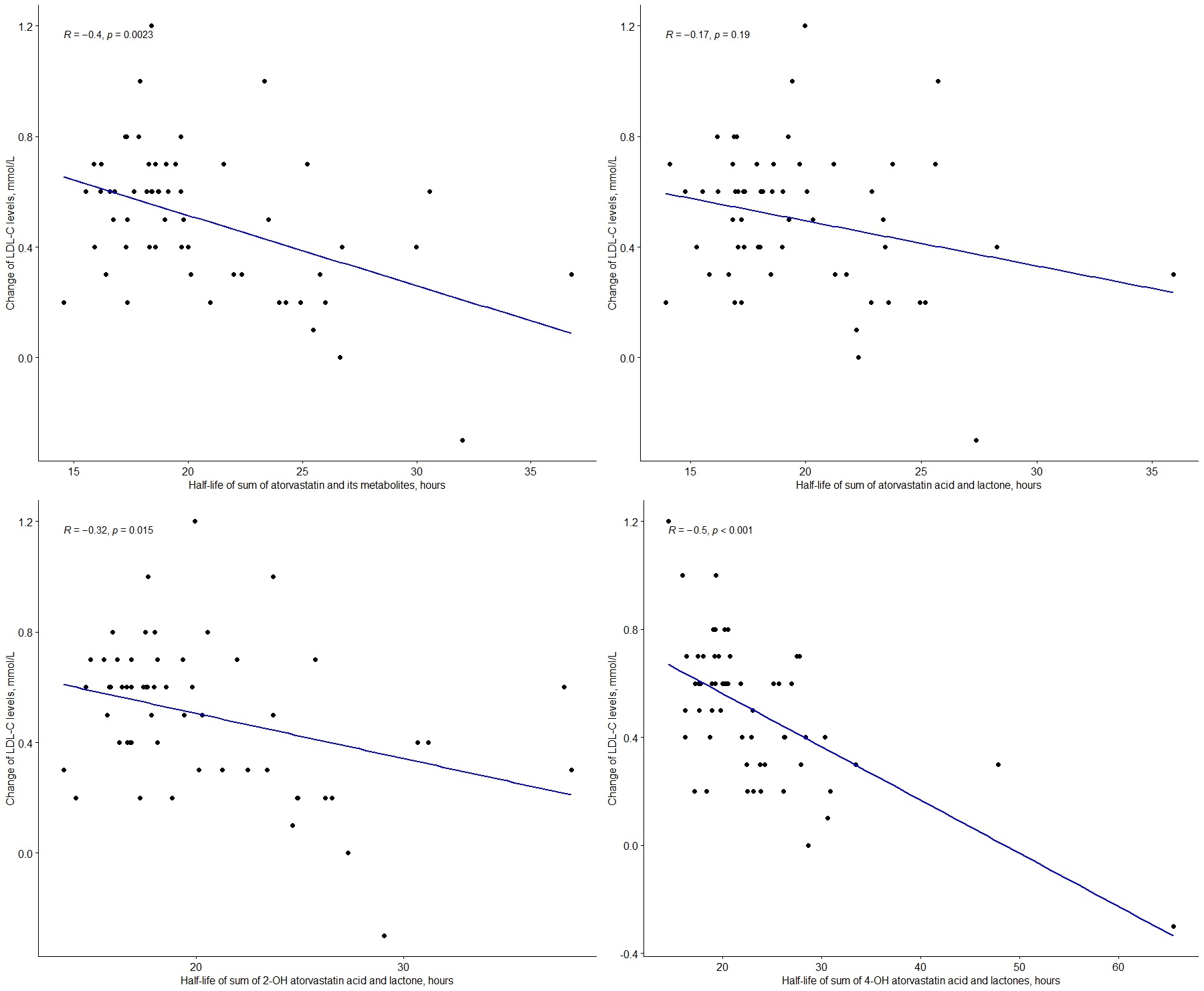

Supplement: Supplementary file 2 — Figure S2. Scatter plots with correlation of low‐density lipoprotein cholesterol concentrations and half‐lives of different atorvastatin metabolites In each scatter plot the black dot indicates the relationship of individual observation, the regression line is a blue line. 2‐OH atorvastatin: ortho‐hydroxyl atorvastatin, 4‐OH atorvastatin: para‐hydroxyl atorvastatin, R: Spearman’s rank correlation coefficient. [file PRP2-13-e70082-s002.jpeg]
